# Anatomical Study of the Hepatic Veins in Segment 4 of the Liver Using Three-Dimensional Visualization

**DOI:** 10.3389/fsurg.2021.702280

**Published:** 2021-08-03

**Authors:** Jun Zhang, Xiaochao Guo, Qilu Qiao, Jianxun Zhao, Xin Wang

**Affiliations:** ^1^Department of General Surgery, Peking University First Hospital, Beijing, China; ^2^Department of Imaging, Peking University First Hospital, Beijing, China

**Keywords:** three-dimensional visualization, anatomical structure, segment IV hepatic veins, drainage, preoperative planning

## Abstract

**Objective:** The current study aimed to examine the anatomical structure of the hepatic vein of segment IV liver (S4) of the liver using three-dimensional (3D) visualization technology in order to explore the surgical value of the middle hepatic vein (MHV) manipulation and highlight the importance of current research in hepatic surgery.

**Methods:** Between January 2014 and December 2019, 52 patients with abdominal diseases(not including hepatic disease) were selected for multiphasic computed tomography-enhanced scans of the upper abdomen. A 3D visualization system was utilized to display the structural details of the hepatic veins in S4 of their livers. Couinaud's eight-segment classification system was used to denote the liver' sections.

**Results:** The constructed 3D model clearly displayed vascular morphological characteristics and their location in the liver, hepatic artery and vein system, and portal vein system. Of the 52 patients, 43 had an umbilical fissure vein (UFV) (82.7%), 19 had an accessory S4 liver vein (36.5%), 16 had both a UFV (30.8%) and an accessory S4 liver vein, and 6 had neither (11.5%). A total of 79% of the patients with a UFV and 74.2% of those with an accessory S4 liver vein had venous blood returning into the left hepatic vein.

**Conclusion:** 3D visualization technology was used to determine hepatic venous return of S4 hepatic veins and was found to improve the safety of evaluation in hepatic surgery.

## Introduction

The middle hepatic vein (MHV) is a very important vein in the liver: it returns the venous blood of segments 5, 6, and 8 (S5, S6, and S8) (using Couinaud's eight-segment classification system) (1), and occasionally it drains the venous blood from S6. In addition, part of the hepatic caudate lobe vein flows into the MHV (2, 3). In hepatic surgery, it is still debated whether MHV ligation causes congestion or even necrosis in segment 4 of the liver in cases of a right lobe tumor invading the MHV or during living-donor transplantation of the right half of the liver (4–8), both of which involve the anatomy of S4 hepatic veins and their drainage. Unfortunately, there have been few studies concerning the anatomy of S4 hepatic veins with variant results (9, 10).

At present, three-dimensional (3D) visualization technology can accurately reconstruct the morphological characteristics of each hepatic vascular system. Through rotation, perspective, scaling, and other functions, the positional relationship between each vessel and liver segment can be clearly understood and the origin of each vessel determined. 3D visualization modeling of the liver uses a large number of samples, which reduces the variance of results caused by the limited number of samples in previous anatomical research (11–13). In the present study, 3D visualization technology was used to explore the anatomy of S4 hepatic veins and provide a basis for MHV management in the practice of hepatic surgery.

## Methods

### Subjects

Between January 2014 and December 2019, 52 patients (27 male and 25 female of mean age 55.6 years) were selected for 3D visualization reconstruction in the Peking University First Hospital. All patients with abdominal disease other than a hepatic disease were included. Thus, patients with liver disease were excluded.

The study was conducted in accordance with the Helsinki Declaration (as was revised in 2013). The study was approved by Ethics Committee of the Peking University First Hospital (Clinical Ethics No. 2018-15) and informed consent was taken from all the patients.

### Computed Tomography Scanning

Multiphasic enhanced CT examinations of the upper abdomen were arranged for all patients before surgery. The patient took a supine position in the head–foot direction, and the scanning scope ranged from the diaphragmatic top to the umbilicus, with the whole liver included. Scanning parameters: tube voltage 120 kv; automatic tube current modulation technology (120–300 mas); pitch 0.984; layer thickness 5 mm; scanning field of vision 50 cm, matrix 512 × 512. A Siemens dual-source CT scanner (SOMATOM Definition Flash, Erlangen, Germany) was used. After injection, 20 ml of normal saline was used to flush the tube. The delay time of the scan in the arterial phase was 20–25 s, and that of the venous phase was 55–60 s.

After scanning, the images of each phase were reconstructed in layers of 1 mm, with intervals of 1 mm, and uploaded to the image workstation. The original CT data were saved in DICOM format.

### 3D Reconstruction

All patients' scanned images were stored in DICOM format and imported into a 3D abdomen visualization system (Shenzhen Xudong Co., Shenzhen) for analysis. The original CT data of each phase were segmented and registered by the program. The displayed objects included the liver, the hepatic artery and vein system, and the portal vein system. The liver was segmented according to Couinaud's eight-segment classification system (8). The homogenization, quality-control standards, and 3D visualization model establishment were based on the *Clinical Practice Guidelines for Precision Diagnosis and Treatment of Complex Liver Tumor Guided by Three-Dimensional Visualization Technology* (version 2019) (14).

### 3D Visualization Image Analysis

Only the liver S4s with a diameter of more than 3 mm were used as the main vein for analysis. Deputy chief physicians from the hospital's imaging department and surgical department checked the 3D visualization results together, and the final report was issued when both physicians reached a consensus.

### Statistical Analysis

SPSS v.25 was used for statistical analysis. Continuous variables were represented as mean averages, and classified data were represented as frequency and rate. A descriptive analysis of the constituent ratio was made. The normality of distributions was tested by K-S normal test.

## Results

**Figure 1:**
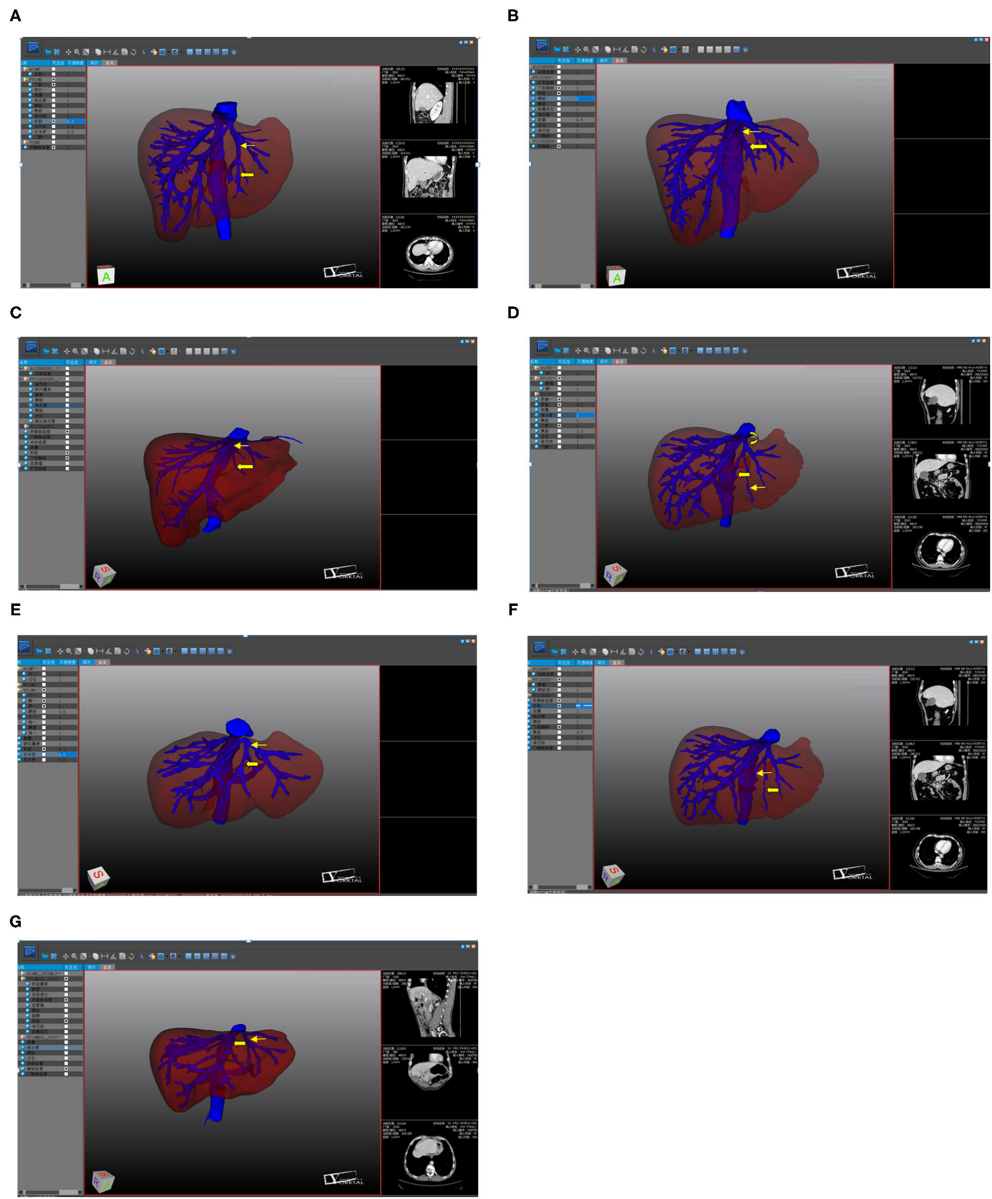
The three-dimensional visualization. **(A)** UFV (Thick arrow); UFV flowing into the LHV (Thin arrow); **(B)** UFV (Thick arrow); UFV flowing into the confluence part of the LHV and the MHV (Thin arrow); **(C)** UFV (Thick arrow); UFV flowing into the MHV (Thin arrow); **(D)** accessory S4 hepatic vein (Thick arrow); UFV (Thin arrow); accessory S4 liver vein flowing into the LHV; **(E)** accessory S4 hepatic vein (Thick arrow); UFV (Thin arrow); accessory S4 hepatic vein flowing into the confluence part of the LHV and the MHV; **(F)** UFV (Thick arrow); accessory S4 hepatic vein (Thin arrow); **(G)** MHV (Thick arrow); LHV (Thin arrow).

Of the 52 patients, 43 (82.7%) had an umbilical fissure vein (UFV). All but one of these cases had S4 branches, accounting for 80.8% of all patients (Figures 1A–C). Nineteen patients (36.5%) had an accessory S4 liver vein (Figures 1D,E), 16 (30.8%) had both a UFV and an accessory S4 liver vein (Figure 1F), and six (11.5%) had neither (Figure 1G). There was no statistical difference between the anatomical morphology of hepatic vein and the general conditions of patients (Table 1).

**Table 1:** Comparison of different anatomical morphology of hepatic vein and the general data of patients.

**Index**	**UFV**	**Accessory S4 liver vein**	**Both a UFV and an accessory S4 liver vein**	**Neither a UFV and an accessory S4 liver vein**	***P*-value**
Age	52.3 ± 2.6	51.4 ± 3.1	51.6 ± 2.1	53.7 ± 2.8	>0.05
Gender					>0.05
Male	21(48.8)	10(52.6)	8(50.0%)	3(50.0%)	
Female	22(51.2)	9(47.4)	8(50.0%)	3(50.0%)	
Height (cm)	167.3 ± 5.6	164.8 ± 4.3	165.2 ± 5.2	166.8 ± 3.6	>0.05
Weight (kg)	60.3 ± 4.8	61.4 ± 3.5	59.6 ± 2.4	59.7 ± 2.8	>0.05

Of the 43 patients with a UFV, 34 (79.0%) had blood returning to the left hepatic vein (LHV), four (9.3%) had blood returning to the MHV, and five (11.6%) had blood returning to the LHV–MHV confluence (see Figures 1A–C). Of the 19 patients with an accessory S4 liver vein, 16 (74.2%) had blood returning to the LHV and three (15.8%) had blood returning to the LHV–MHV confluence (see Figures 1D,E).

## Discussion

With CT and magnetic resonance imaging data and computer image-processing technology, digital 3D visualization technology can analyze, fuse, calculate, segment, and render data. It can also be used to display the bile duct and vascular system separately from the vision intuitively, accurately, and quickly, allowing clinicians to describe and explain the shape and spatial distribution of the bile duct and blood vessels. It can therefore assist in decision making, accurate preoperative diagnosis, individualized surgical planning, and the selection of an appropriate surgical approach (15).

In hepatic surgery, it is crucial to determine the precise location of a tumor before operating, and the control of blood flow in the portal and hepatic veins is an important factor affecting the operation success. The anatomy of the hepatic veins in S4 of the liver plays a role in whether simple removal of the MHV will obstruct blood flow in that segment in patients with right lobular hepatic tumors invading the MHV who cannot undergo a right lobectomy or right hemihepatectomy due to insufficient volume of the residual liver. It is also involved in possible blood flow obstruction in living-donor liver transplantation when the donor liver does not include the MHV. 3D reconstruction of the portal and hepatic veins is therefore of great value in analyzing the morphological features and variations of these veins and can be used when choosing the most appropriate operative plan (15, 16).

In addition to the MHV, the UFV and the accessory S4 liver vein drain the hepatic venous blood in S4 of the liver. Unfortunately, there are very few anatomical studies concerning these two veins, but 3D visualization technology now offers the possibility of a large-sample study of them, thereby improving the safety of hepatectomies and liver transplantations.

The UFV in the *ligamentum teres hepatis*drains the S3 and S4 hepatic veins (1). Some researchers have suggested that the probability of the occurrence of a UFV is <60%; however, the present study found that this probability to be 82.7%, which is much higher than the findings of previous studies. This difference may be because the previous studies were based on the results of autopsies, and the small sample size of such studies is likely to cause selective bias.

In the present study, anS4 branch was found in 97.7% of all patients with an UFV, which is slightly higher than the reported 93% of previous studies (17–21). It has also been previously reported that 90% of UFVs flow into the LHV, 7% into the MHV, and 3% into the LHV–MHV confluence (22). However, the 3D visualization reconstruction in the present study found that 79% of UFVs flowed into the LHV, 11.6% into the LHV–MHV confluence, and 9.3% into the MHV. It was also noted that the distance between the UFV inlet and the MHV root did not exceed 1 cm. These results indicate that the occurrence of UFV is widespread and that, in most cases, the UFV enters the LHV or the LHV–MHV confluence. Even when the UFV enters the MHV, it only enters within 1 cm of the root of the MHV. Therefore, as long as ligation is not performed too close to the root of the MHV during hepatic surgery, the blood return of the UFV will not be obstructed.

The accessory S4 liver vein is not the S4 branch of the MHV but a hepatic vein running alone in S4 of the liver and eventually flowing into the LHV or the LHV–MHV confluence. In the 3D visualization conducted in the present study, 36.5% of the patients were found to have an accessory S4 liver vein, which suggests that this vein might be common in the general population. The presence of this vein was also not found to be related to gender.

In the 52 patients, 16 cases had both UFV and S4, accounting for 30.8%. Six cases had neither. This indicates that MHV ligation will not affect the blood flow in S4 of the liver in most patients—or at least not cause an overall blood-flow disorder in the hepatic veins in the segment.

For most patients, the return of the hepatic vein in segment IV of the liver relies on the other hepatic venous return systems in addition to on the MHV. Therefore, ligation of the MHV does not affect the return of blood to segment IV liver.

## Conclusion

Wing to the maturity and vigorous development of 3D visualization technology, Great progress has been made in the anatomical reconstruction of important hepatic ducts. The anatomy of hepatic vein of segment IV liver scan be accurately and comprehensively analyzed. This analysis provides a reference for the precise hepatectomy of S4 of the liver and can offer sufficient preoperative evaluation for liver transplantation, thereby improving the safety assessment of hepatic surgery

## Data Availability Statement

The original contributions presented in the study are included in the article/supplementary material, further inquiries can be directed to the corresponding author/s.

## Author Contributions

QQ: conception and design of the research. JZhan and JZhao: acquisition of data and critical revision of the manuscript for intellectual content. XG and XW: analysis and interpretation of the data. JZhan and QQ: statistical analysis. XG and XW: writing of the manuscript. All authors contributed to the article and approved the submitted version.

## Conflict of Interest

The authors declare that the research was conducted in the absence of any commercial or financial relationships that could be construed as a potential conflict of interest.

## Publisher's Note

All claims expressed in this article are solely those of the authors and do not necessarily represent those of their affiliated organizations, or those of the publisher, the editors and the reviewers. Any product that may be evaluated in this article, or claim that may be made by its manufacturer, is not guaranteed or endorsed by the publisher.
